# Touch-stimulation increases host-seeking behavior in *Steinernema Carpocapsae*

**DOI:** 10.21307/jofnem-2019-067

**Published:** 2019-10-14

**Authors:** Tiffany Baiocchi, Lauren Braun, Adler R. Dillman

**Affiliations:** 1 Department of Nematology, University of California Riverside, Riverside, CA, 92521

**Keywords:** *Steinernema*, Host-seeking behavior, Chemotaxis

## Abstract

Previous research demonstrated that *Steinernema carpocapsae* infective juveniles (IJs) exposed to a host cuticle were more attracted toward certain host-associated volatile odors. We wanted to test the specificity of attraction that results from exposure to host cuticle. Host recognition behavior was analyzed after stimulating IJs by allowing them to physically interact with *Galleria mellonella* cuticles. The subsequent behavioral response and the proportion of the population participating in chemotaxis to multiple host odors were measured. We found that exposure to host cuticles resulted in a significantly higher percentage of the population participating in host-seeking behavior, with threefold more nematodes participating in chemotaxis. We tested whether exposure to live or dead host cuticle resulted in a different response and found that a higher percentage of IJs exposed to a live host cuticle participated in chemotaxis than IJs exposed to a dead host cuticle, but that IJs exposed to a dead host demonstrated significantly higher participation than was observed for non-stimulated IJs. To test whether the increase in IJ participation in host-seeking behaviors after exposure to a live host cuticle was specific, we exposed stimulated IJs to a known repulsive odor, a neutral odor, and two predicted attractants. We found that stimulation of IJs through physical contact with a host cuticle induces a specific enhancement of host-seeking behavior to host-specific odors rather than a general increased chemotactic response to all volatile stimuli. However, the nematodes displayed an enhanced response to multiple host-specific odors. Future work should focus on the mechanism through which contact with live host cuticle stimulates increased behavioral response.

Entomopathogenic nematodes (EPNs) are a guild of insect-parasitic nematodes that are used in biological control to kill insect pests and prevent crop loss due to insect herbivory (Kaya and Gaugler, 1993; Dillman and Sternberg, 2012; Lewis and Clarke, 2012). Infective juveniles (IJs) are modified third-stage larvae, analogous to dauer larvae in *C. elegans*. The IJs are the only free-living stage in the EPN life cycle and they carry mutualistic entomopathogenic bacteria as they seek out and infect a new insect host. IJs invade the host – releasing their symbolic bacteria in the infection process – and resume development to complete their life cycle (Wright and Perry, 2002). The IJs are the only stage found outside the host, and the only stage known to participate in host-seeking behavior. The success of EPNs in biological control is dependent on a variety of characteristics including virulence, stress tolerance, and behavioral traits such as dispersal and host-seeking behavior. The host-seeking behaviors of EPNs vary along a continuum and differ between species. One endpoint along this continuum is categorized as cruise-foraging; cruisers actively search for sedentary hosts in the soil (Campbell and Gaugler, 1997; Lewis, 2002b; Lewis et al., 2006). The other endpoint on the foraging continuum is an ambush strategy where the IJs stay in one place and wait for a host to pass by before attaching to the host and infecting it. Species of EPNs have been categorized as ambush, cruise, or intermediate foragers based on several characteristics including their mobility and whether or not they can tail-stand (Lewis et al., 1992; Campbell and Gaugler, 1993; Lewis et al., 1993; Campbell and Gaugler, 1997). *Steinernema carpocapsae*, which can stand upright on its tail, jump, tail-stand, and has low mobility has been classified as an ambush forager (Campbell and Gaugler, 1997; Bal et al., 2014).

The majority of *S. carpocapsae* IJs do not actively engage in host-seeking chemotaxis behavior, even in the presence of host odors (Gaugler et al., 1990; Lewis et al., 1992; Campbell and Gaugler, 1993; Lewis et al., 1995, 1996; Bal et al., 2014; Baiocchi et al., 2017). *S. carpocapsae* IJs spend a great deal of their foraging time performing tail standing – which lifts them from the substrate and allows them to perform jumping behavior – however, their foraging behavior can vary with their environment (Wilson et al., 2012; Kapranas et al., 2017; Hiltpold and Hibbard, 2018). Ambush foragers, which are effective at finding and infecting active hosts may come into brief physical contact but fall off or be separated from the host due to behavioral immunity such as grooming or rasping behavior of the host (Grewal et al., 1994; Lewis et al., 1995; Campbell and Kaya, 2002; Lewis et al., 2006). In such cases, having physical contact with the host was hypothesized to lead to some alteration in host-seeking behavior, such as localized search after contact. It was demonstrated that after stimulation by physical contact with host cues, a strikingly higher proportion of *S. carpocapsae* IJs participate in chemotaxis behavior toward host-associated cues (Lewis et al., 1995, 1996). For naïve *S. carpocapsae* IJs, only ~4 to 25% of the population participate in chemotaxis behavior toward host volatiles, ~45 to 60% of IJs stimulated by physical contact with the host participate in chemotaxis (Lewis et al., 1995, 1996).

Our objective was to further characterize the behavioral shift of *S. carpocapsae* IJs after physical contact with potential hosts and to determine whether their increased participation in host-seeking behavior was a specific response to a particular host, or a general increase in localized search behavior. We hypothesized that *S. carpocapsae* IJs stimulated by physical contact with a potential host would be generally more responsive to environmental stimuli.

## Materials and methods

### Nematode culturing

*S. carpocapsae* was from the inbred strain All – using standard procedures (Kaya and Stock, 1997). Last-instar *Galleria mellonella* were placed in a 6 cm petri dish and infected with approximately 30 nematodes per host. They were then incubated at room temperature (approximately 23°C) for 7 to 10 d. After this incubation period, infected and deceased hosts were placed on white traps, which were incubated at room temperature for another 7 to 10 d. IJs were collected from the white traps, rinsed three times with tap water, and placed within cell culture flasks. A portion of these IJs were used immediately after collection for our ‘no-exposure’ control assays, while the rest were exposed to host cuticles and used for chemotaxis assays within the hour.

### Exposure to host cuticle

For exposure to live host cuticle, mesh paper was placed onto a filter fitted onto a 2 L Erlenmeyer vacuum filtration flask. Approximately 250 μL of IJs were placed onto the filter several times until a mass of IJs was visible. A waxworm (*Galleria mellonella*) was then placed onto the IJs and manually rolled through them. The waxworm was then placed into a 10 cm petri dish with a dampened piece of 10 cm filter paper to ensure that the IJs did not dry up and die while on the waxworm cuticle. Nine more waxworms were exposed to the IJs and all were placed into the same petri dish as the first one. These waxworms were incubated at room temperature for 30 min to ensure that all of the IJs made contact with the host cuticle. After 30 min, the waxworms were placed into a 50 mL conical and rinsed with 15 mL of tap water. The conical was gently shaken to dislodge any IJs attached to the waxworm cuticles, and the water was pipetted out and transferred to a 15 mL conical. These IJs were spun down in a centrifuge and rinsed three times. After the third rinse, the IJs were transferred into culture flasks and quantified to find the concentration of IJs. IJs were then immediately used for behavioral (chemotaxis assays).

In total, 10 to 12 live *G. mellonella* were transferred onto a 10 cm petri dish and placed into a −20°C freezer for 30 min. After this time, the plate was removed and left at room temperature for another 30 min to defrost the hosts. These freeze-killed hosts were used in the same methods described above for the live host cuticle exposures.

### Chemotaxis to host volatiles

Chemotaxis media plates were prepared as previously described and allowed to sit at room temperature for a minimum of 12 hr before being used (Hallem et al., 2011; Dillman et al., 2012; Baiocchi et al., 2017). In total, 50 mL Hamilton gas-tight syringes were used; test syringes were filled with five live, non-infected *G. mellonella* larvae, while the control syringes were left empty. The syringes were then loaded into a KD Scientific pump (Model: KDS 220, Catalog No. 78-0220NLSU).

Petri dish lids from 10 cm plates were modified. Two 10 mm holes were drilled on either side of the lid approximately 10 mm away from the edges. Nalgene PVC tubing (1/8′′ diameter) was attached into these holes and onto the syringes to connect the two. This tubing allowed air from the syringes to travel onto the scoring circles, which were attached to the bottom of the chemotaxis plate. A pellet of approximately 250 μL IJs were placed onto the center of the chemotaxis plate. The modified lids were placed onto the plates and positioned so that the tubing rested above its corresponding scoring circle. The plates were set on a vibration-reducing platform for the duration of the assays. The assays ran for approximately 1 hr and 10 min. The assays were scored with the scoring template attached to the bottom of the chemotaxis plates. A minimum of three plates were run for each experiment for each time point, and each experiment consisted of nine technical replicates. One full experiment was run with IJs not exposed to the host cuticle, while another full experiment was run with exposed IJs.

Chemotaxis index (CI) values were determined by counting the number of IJs within each scoring circle, which were being exposed to our host odors and control. CI was calculated using this equation: CI = No. in host circle – No. in control circle/sum of all individuals within both circles.

We determined participation by counting all IJs that had moved at least 1 cm away from the center. Those that did not move that distance were designated as remaining in the middle section.

### Chemotaxis to soluble odors

Chemotaxis media plates were prepared as previously described and allowed to sit at room temperature for a minimum of 12 hr (Hallem et al., 2011; Dillman et al., 2012; Baiocchi et al., 2017). A total of 2 μL of sodium azide (NaN_3_) was placed in each scoring circle to act as a paralytic agent. Immediately after placing the sodium azide, 5 μL of our test chemical was placed on top of the test scoring circle, followed by 5 μL of our control chemical (water or ethanol) being placed on the control scoring circle. A 5 μL pellet of IJs was placed into the center of the chemotaxis template, which was located on the bottom of the chemotaxis plates. These plates were then stacked into groups of three and placed in a box with a lid and put onto a vibration resistant platform for the remainder of the assay run. We let the assays run for 1 hr and 10 min to ensure that enough IJs had dispersed properly. Chemotaxis index and participation were calculated as described above. A minimum of three plates were run for each experiment for each time point, and each experiment consisted of nine technical replicates. One full experiment was run with IJs not exposed to the host cuticle, while another full experiment was run with exposed IJs.

### Statistical analysis

All statistical analyses were conducted using GraphPad Prism software. Chemotaxis index statistical analyses were done using unpaired, one-way Anova testing. Participation was examined by analyzing the date points within the host, middle, and control sections. Analysis was done using unpaired, two-way Anova testing.

## Results

**Figure 1:**
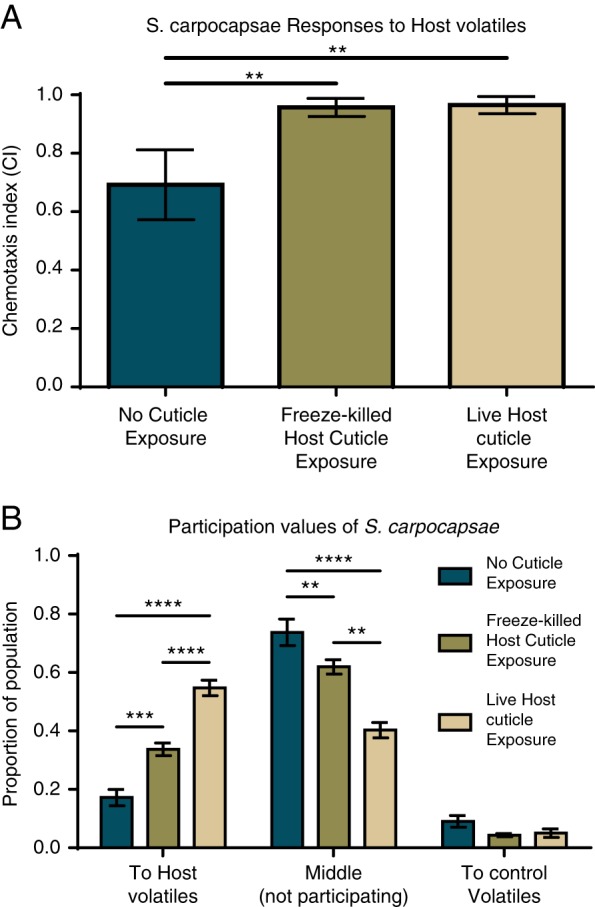
(A) Chemotaxis assays performed on *S. carpocapsae* IJs after exposure to live and freeze-killed host cuticles, where +1 indicates strong attraction, near 0 indicates indifference, and −1 indicates strong repulsion. (B) Participation evaluation of *S. carpocapsae* IJs after exposure to live and freeze-killed host cuticles, where ‘to host volatiles’ indicates attraction, ‘middle’ indicates indifference, and ‘to control’ indicates repulsion toward our test volatile. Statistical significance was determined with an unpaired, one-way ANOVA test for CI, and two-way ANOVA testing for participation. Error bars represent SEM. ** *P* < 0.01; *** *P* < 0.001; **** *P* < 0.0001.

We evaluated the effects of exposing *S. carpocapsae* infective juveniles (IJs) to the cuticle of live waxworms and the cuticle of freeze-killed waxworms. We found that, as previously reported, naïve IJs have low participation in chemotaxis host-seeking behavior, with approximately ≤20% of the population engaging in chemotactic behavior in the presence of host volatiles (Fig. 1) (Lewis et al., 1992; Campbell and Gaugler, 1993; Lewis et al., 1995; Lewis et al., 1996; Bal et al., 2014; Baiocchi et al., 2017). The IJs that do chemotax demonstrate strong attraction toward waxworm odors (Fig. 1). We found that exposure to live waxworm cuticle or freeze-killed waxworm cuticle resulted in both increased attraction to waxworm volatile odors as well as increased participation in host-seeking behavior (Fig. 1). The increased participation resulting from contact with live waxworm cuticle is striking and led to >50% of the IJ population participating in host-seeking behavior (Fig. 1B).

**Figure 2:**
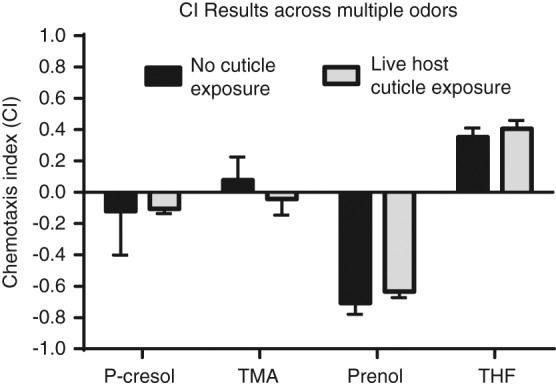
The chemotaxis index of *S. carpocapsae* IJs responding to four host-associated chemicals, where +1 indicates strong attraction, 0 indicates indifference, and −1 indicates strong repulsion. Statistical significance was determined with an unpaired, ordinary, one-way ANOVA test. Error bars represent SEM.

Next, we wanted to determine whether the increased attraction to host-associated odors was specific or the result of a general increase in responsiveness to environmental odors, and whether the behavioral response valence to attractive, neutral, or repulsive odors had changed (Hallem et al., 2011). Therefore, we exposed naïve IJs and IJs that had been stimulated by contact with live waxworm cuticle to four odors; p-cresol (4-methylphenol), prenol, tetrahydrofuran (THF), and trimethylamine. The odors p-cresol and THF are associated with mole crickets and earwigs, respectively, and are reported attractants of *S. carpocapsae* IJs (Dillman et al., 2012). Trimethylamine (TMA) is associated with house crickets and pillbugs and is reportedly a neutral odor, neither attractive nor repulsive to *S. carpocapsae* IJs (Dillman et al., 2012). Prenol was recently identified as being associated with waxworms infected by EPNs and elicits a repulsive response from *S. carpocapsae* IJs (Baiocchi et al., 2017). We found that stimulating IJs by physical contact with waxworm cuticle did not significantly affect the chemotactic behavioral response or valence to any of the four host-associated odors – which were tested individually (Fig. 2). In our assays, p-Cresol and TMA were neutral or near neutral for naïve and stimulated IJs (Fig. 2). Prenol was strongly repulsive and THF was strongly attractive to both naïve and stimulated IJs as previously reported (Dillman et al., 2012; Baiocchi et al., 2017), with no significant difference in chemotaxis index between the IJ populations.

**Figure 3:**
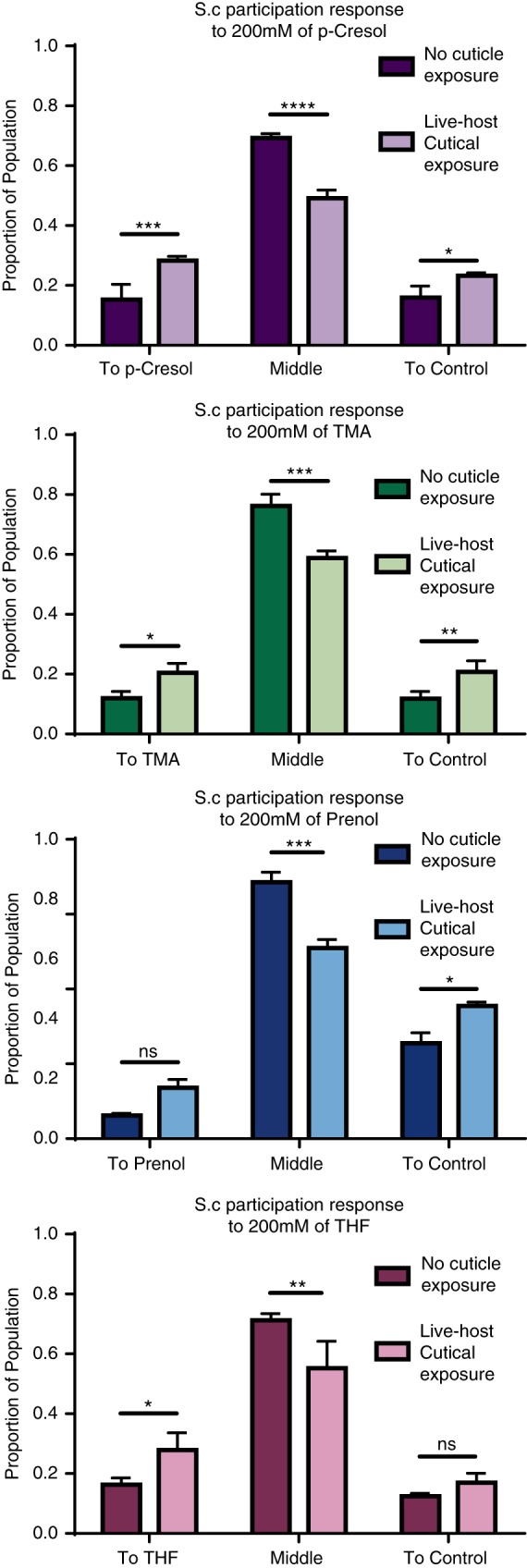
Graphs of IJ participation in response to p-Cresol (4-methylphenol) (A), Trimethylamine (B), Prenol (C), and Tetrahydrofuran (D). We tested IJs exposed to live host cuticles and non-exposed IJs. The IJs either traveled toward the test volatile, stayed in the middle region, or moved away from the test volatile and toward the control.

After measuring the behavioral response to specific host odors using a chemotaxis index, we evaluated the effect of exposure to live waxworm cuticle on the participation of *S. carpocapsae* IJs in host-seeking behavior. We found that for all odors – neutral, repulsive, and attractive – the proportion of the *S. carpocapsae* IJ population participating in host-seeking behavior increased significantly. In response to p-Cresol and TMA, the number of IJs migrating away from the point of initial placement either toward the odorant or away from the odorant increased when they had been exposed to waxworm cuticle (Fig. 3). Significantly more IJs stimulated by waxworm cuticle moved away from the repulsive odor prenol than naïve IJs, and significantly more IJs stimulated by waxworm cuticle moved toward the attractive odor THF (Fig. 3).

## Discussion

Exposure to stimuli – such as temperature (Lee et al., 2016), volatile chemicals (Willett et al., 2015; Willett et al., 2017), magnetic fields (Ilan et al., 2013), or touch (Campbell and Kaya, 2000; Lewis, 2002a; Goodman, 2006) – have been shown to elicit behavioral shifts in a variety of organisms including EPNs. Exposure to certain cues has even been shown to have multi-trophic consequences, including improvement of the probability of successful infection of insects by EPNs (Willett et al., 2017). Generally, host-seeking behaviors of EPNs fall along the spectrum between ambusher and cruiser foraging strategies (Campbell and Lewis, 2002; Lewis, 2002b), and in this study we have investigated host-seeking by *S. carpocapsae*, the canonical and most well-studied ambush-foraging EPN. Part of what defines *S. carpocapsae* as an ambush forager is that in addition to crawling by sinusoidal movement on substrate, it exhibits standing and jumping behaviors, which are not performed by cruise-foraging EPNs (Campbell and Lewis, 2002; Lewis, 2002a; Lewis et al., 2006). Furthermore, unlike intermediate- and cruise-foraging EPNs, naïve IJs exhibit low participation in chemotaxis behavior to odorants, with ~5 to 25% of naïve *S. carpocapsae* IJs reportedly participating in chemotaxis behavior when exposed to mixtures of host-associated odors or individual host-associated odors (Lewis et al., 1995, 1996; Bal et al., 2014; Baiocchi et al., 2017). We were intrigued by previous studies, which had shown that physical contact with host cuticle had a striking effect on *S. carpocapsae* IJs, leading to a significant increase in the proportion of IJs participating in chemotaxis host-seeking behavior (Lewis et al., 1995, 1996). We have replicated those results and confirm that physical stimulation by host cuticle increases the proportion of *S. carpocapsae* IJs that participate in chemotaxis. This raised the question: why respond to host volatiles only after attachment or contact with a host? Previous researchers hypothesized that because *S. carpocapsae* had been reported to preferentially enter hosts via the spiracles (Georgis and Hague, 1981), short-range attraction to volatiles emanating from the spiracles such as CO_2_ would facilitate invasion of the host (Lewis et al., 1996). Our results suggest that stimulation by physical contact with host cuticle increases IJ participation in chemotaxis to a variety of odorants, not just attractants but also repellants and neutral cues as well. While we have found that stimulated IJs have enhanced participation in host-seeking behavior, our understanding of this process remains limited. How long after physical stimulation does the effect last? What neurological and molecular processes underlie this dramatic change? Future studies could use single-nematode RNA-seq to determine changes in transcriptional profiles associated with stimulation by host cuticle (Serra et al., 2018), and perform functional studies to validate the role of certain neurons and genes that play a role in this change in behavior (Hallem et al., 2011; Morris et al., 2017).

Infection by EPN is hypothesized to follow the hierarchical steps of host habitat location, host location, host acceptance, and host suitability (Campbell and Lewis, 2002). Many studies, including ours, have focused on the host-location or host-seeking step of the infection process, investigating how EPN IJs locate hosts. While EPNs have been shown to use a variety of sensory modalities including thermoreception (Lewis, 2002a), mechanoreception (Torr et al., 2004), magnetoreception (Shapiro-Ilan et al., 2009; Ilan et al., 2013), and electroreception (Ilan et al., 2013); most studies have focused on chemoreception (Pye and Burman, 1981; Hallem et al., 2011; Gang and Hallem, 2016). The relative importance of these modalities in EPN host-seeking is not clear, though one study argues that mechanoreception is more important than chemoreception (Torr et al., 2004). More research on the role of mechanoreception and its relative importance in EPN host-seeking would help inform future studies.
